# SPH simulations and experimental investigation of water flow through a Venturi meter of rectangular cross-section

**DOI:** 10.1038/s41598-023-48520-8

**Published:** 2023-12-01

**Authors:** Leonardo Di G. Sigalotti, Carlos E. Alvarado-Rodríguez, Fernando Aragón, Valeriano S. Álvarez Salazar, Ignacio Carvajal-Mariscal, Cesar A. Real Ramirez, Jesus Gonzalez-Trejo, Jaime Klapp

**Affiliations:** 1 Departamento de Ciencias Básicas, Universidad Autónoma Metropolitana - Azcapotzalco, Mexico City, 02128 Mexico; 2 Departamento de Ingeniería Química, Universidad de Guanajuato, Guanajuato, 3605 Mexico; 3 Consejo Nacional de Humanidades, Ciencias y Tecnologías, Mexico City, 03940 Mexico; 4 Departamento de Energía, Universidad Autónoma Metropolitana - Azcapotzalco, Mexico City, 02128 Mexico; 5 ESIME UPALM, Instituto Politécnico Nacional, Mexico City, 07738 Mexico; 6 Departamento de Sistemas, Universidad Autónoma Metropolitana - Azcapotzalco, Mexico City, 02128 Mexico; 7 Departamento de Física, Instituto Nacional de Investigaciones Nucleares, La Marquesa, 52750 Mexico

**Keywords:** Engineering, Mathematics and computing

## Abstract

The flow of water through a horizontal small-scale Venturi tube of rectangular cross-section is simulated using a modified version of the open-source code DualSPHysics, which is based on Smoothed Particle Hydrodynamics (SPH) methods. Water is simulated using the Murnaghan-Tait equation of state so that weak compressibility is allowed. The hydrodynamics is coupled to a Large-Eddy Simulation (LES) turbulence model. The convergence properties of SPH are improved by adopting a C$^{2}$ Wendland function as the interpolation kernel, increased number of neighboring particles and non-reflective open boundary conditions at the outlet of the Venturi tube. The flow structure and differential pressure as well as the mainstream velocity profiles at different stations are compared with calibrated experimental data. A resolution independence test shows that good convergence to the experimental measurements is achieved using four million particles. At this resolution the simulations predict the experimental centerline velocity profile along the Venturi meter for a volumetric flow rate of ten liters per minutes (lpm) with a root-mean-square error of 4.3%. This error grows to 7.1% when the volumetric flow rate increases to 25 lpm. The predicted differential pressure matches the experimental data with errors varying from 1.4% (for 10 lpm) to 6.8% (for 25 lpm). Cross-sectional velocity profiles within the throat and divergent sections differ from the experimental measurements in less than 5.5%. In general, it is shown that the SPH model can provide an efficient and accurate method for recalibrating flow meters at moderately high Reynolds numbers instead of using costly experimental tests.

## Introduction

Venturi tubes are obstruction meters employed in many industrial applications and laboratory experiments to measure the volumetric flow rate of a fluid in a pipe. A typical Venturi meter is a tubular device of varying cross-sectional area, consisting of a convergent conical section that leads to a constricted pipe section, or throat, which is then followed by a divergent conical section, or diffuser, in which the diameter increases again to that of the main pipeline^1^. When the fluid enters the convergent section, its velocity increases as the pipe diameter decreases, resulting in an effective pressure drop according to Bernoulli’s equation^2^. This differential pressure across the convergent-divergent sections is considered to be the most important parameter to determine the performance of the Venturi tube^3^. This pressure reduction is called the Venturi effect^4^.

Over the past decades, Venturi tubes have been widely used as wet scrubbing systems in numerous industrial applications and in nuclear power plants to remove particles and gaseous pollutants from waste gas streams prior to their emission into the environment^5–9^. In addition to being one of the most popular and highly efficient gas cleaning devices, Venturi injectors have also found important applications in sprinkler and drip irrigation systems for intensive farming and industrial agriculture^10^. They have also been used in applications of liquid flow metering^11^, mixing of liquid/liquid and gas/liquid systems as, for example, in the injection of ozone in water and in water aeration systems^12–14^, and in the enhancement of the chemical reaction of hydrodynamic cavitating flows^15^. Efforts have also focused on studying the instability of cavitating flows in a Venturi meter, whose control can help to limit structural damage^16^. In general, Venturi injectors operate over a wide range of pressures, are simple in design, have no moving parts and are almost free of maintenance. Due to all these advantages and their widespread applications in the industry, a wide range of models of varying complexity have been presented in the literature, which have been frequently validated experimentally.

In addition to the large number of existing experiments, a great deal of effort has also been devoted to the numerical modeling of Venturi flows in order to reproduce the experimental data for laboratory-scale and industrial-size devices. Theoretical studies on the hydrodynamics of Venturi meters range from simple correlations to more complex numerical simulations. For instance, a boundary layer growth model was developed by Gamisans et al.^17^ to investigate the pressure drop characteristics of Venturi scrubbers, while the hydrodynamics of a liquid/liquid phase distribution through a Venturi meter in a vertical pipe was examined by Jana et al.^18^. Numerical simulations of flow behavior within a Venturi meter have been performed to determine air injection rates^19^, pressure differences on a bluff body object for water flow^20^ and on turbine blades placed within the throat section for airflow^21^, as well as to determine the effects of varying the geometry of the convergent section on pressure drop^22^ and study the behavior of two-phase flow and mixing^23–27^. More recently, cavitating flows in a small-sized Venturi tube have been studied by Razali et al.^28^ Further numerical simulations have been carried out to measure discharge coefficients and pressure drops in industrial-sized Venturi meters for oxygen-free nitrogen^29^ and water flow^30^. The effects of varying the structural parameters of a Venturi tube, including the contraction ratio, the ratio of the throat section length to diameter, the diffusion angle and the inlet and outlet pressure difference, on fluid flow were analyzed by Zhang^31^ using the Fluent v6.3 software. Finite element methods have been used for design optimization analysis of a Venturi tube for medium conveying in strengthen grinding processes^32^, while investigation of large over-reading of the Venturi flow rate in the feed water flow control system in the second circuit of a nuclear power plant was recently performed by Wang et al.^9^ with the aid of the software package ANSYS Fluent 17.0.

Most early and recent simulations of the Venturi flow have been carried out using traditional mesh-dependent methods and no calculations have been performed to date using the method of Smoothed Particle Hydrodynamics (SPH). In this study, we perform three-dimensional calculations of water flow at Reynolds numbers between 13272 and 33180 through a small-scale Venturi tube of rectangular cross-sectional shape, using a modified version of the open-source code DualSPHysics^33^, which is based on SPH theory for solving the equations of fluid dynamics. Water is simulated using the Murnaghan-Tait equation of state so that a weakly compressible SPH scheme is proposed^34^. The Large-Eddy Simulation (LES) turbulence model is implemented to filter the equations of fluid dynamics. The convergence properties of SPH are improved in three different ways. First, a C$^{2}$ Wendland function^35,36^ is used as the interpolation kernel. In contrast to most conventional kernels, Wendland functions support large numbers of neighboring particles without becoming unstable and do not allow particle motions on a sub-resolution scale (i.e., within the kernel support). Therefore, they maintain quasi-ordered particle distributions even in highly dynamical test problems. Second, approximate volume partitioning is achieved using a large number of neighbors^37,38^. Third, non-reflecting outlet boundary conditions are implemented by allowing the particles that leave the Venturi diffuser and enter the outflow zone to move according to an outgoing wave equation for the velocity field. This effectively reduces feedback noises from the outlet boundary, improving the accuracy of the solution. The Venturi tube was designed for experimental purposes and the numerically obtained flow behavior is compared with calibrated experimental flow measurements. The motivation for this study is twofold. First, understand the flow measurement device and second, explore the efficiency and accuracy of the proposed SPH method for recalibrating flow meters at moderately high Reynolds numbers. In particular, the flow structure along the convergent, throat and divergent sections of the Venturi as well as the differential pressure and mainstream velocity profiles at different stations along the tube are compared with the experimental data. The results show that the experimental measurements are reproduced with errors less than about 7.1% in the velocity profiles (when measured in units of m s$^{-1}$) and less than $\sim 6.8$% in differential pressure (in units of psi) along the length of the Venturi for the highest flow rates considered.

**Figure 1:**
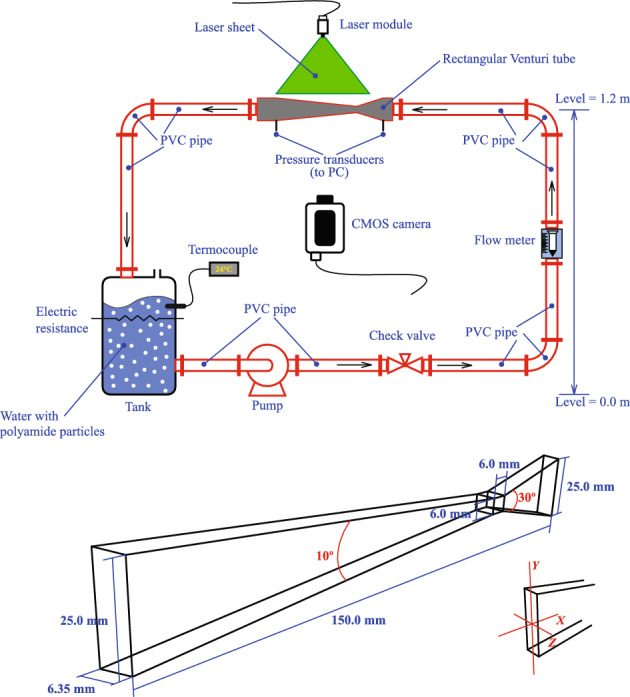
Schematic of the flow test loop used for the experiments (top) and three-dimensional view and description of the small-sized Venturi flow meter (bottom).

## Materials and methods

### Experimental flow loop

The experimental flow measurements were performed using a closed flow loop as shown schematically in the upper part of Fig. 1. The Venturi tube is installed horizontally with the differential pressure and has a rectangular cross-sectional area as depicted in the lower part of Fig. 1. It is composed of a convergent section of length 35.45 mm and convergent angle of $30^{\circ }$, a throat of length 6 mm and a diffuser of length 108.57 mm and divergent angle of $10^{\circ }$. The cross-sectional area at the inlet of the convergent section and outlet of the diffuser is $a\times b=6.35\times 25$ mm$^{2}$, while the throat section has a cross-sectional area of $a\times b=6\times 6.35$ mm$^{2}$. The inlet and outlet planes of the Venturi meter have hydraulic diameters, $D_{\text{H}}=2ab/(a+b)\approx 10.13$ mm, while the throat has a hydraulic diameter of $\approx 6.17$ mm. For this meter, the contraction ratio, calculated as the ratio between the height of the throat and that of the inlet plane, is $\gamma =6/25=0.24$.

**Figure 2:**
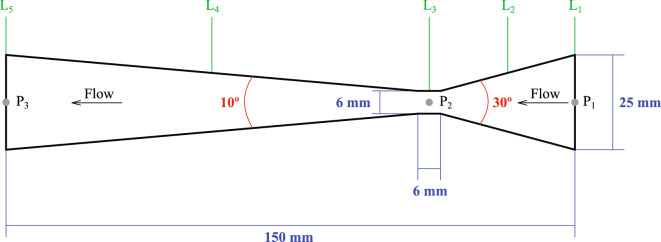
Schematic side view of the Venturi meter. P$_{1}$, P$_{2}$ and P$_{3}$ mark the points where pressure values were measured, while L$_{1}$, L$_{2}$, L$_{3}$, L$_{4}$ and L$_{5}$ mark the stations along the Venturi where mainstream cross-sectional velocity profiles were measured experimentally.

The Venturi meter is made of transparent acrylic to facilitate flow visualization, while the pipeline upstream and downstream of the Venturi is made of PVC and has a circular cross-section of diameter 25 mm. The procedure undertaken in the experiments is as follows. First, a peripheral pump of 1 HP capacity is switched on to drive water from a 50-liter storage tank open to the atmosphere through an orifice. The flow is controlled by a check valve, which is placed upstream of a flow meter which in turn adjusts the volumetric flow rate at the inlet of the Venturi to be in the range between 10 and 35 liters per minute (lpm). When the circuit is completely filled with water, the pump keeps the circulation going by maintaining a head difference and the desired flow. The dynamic pressure is measured by means of three pressure transducers placed at the inlet (P$_{1}$), in the middle of the throat (P$_{2}$), and at the outlet plane (P$_{3}$) (see Fig. 2). The characteristics of the flow were determined using the particle image velocimetry (PIV) technique, which enables an accurate spatial and temporal resolution of the velocity field. A centerline pressure profile along the length of the Venturi tube is determined by applying Bernoulli’s equation

$$\begin{aligned} p_{j+1}=p_{j}-\frac{1}{2}\left( \rho _{j+1}v_{j+1}^{2}-\rho _{j}v_{j}^{2} \right) , \end{aligned}$$

for $j=1,2,\cdots ,M-1$, where for $j=1$ the value of $p_{j}$ is given by the experimentally measured pressure at point P$_{1}$, the values of $v_{j}$ correspond to the measured mainstream flow velocities along the Venturi meter, and *M* stands for the number of points on the centerline where measured values of the velocity are known. This procedure is repeated by iterating backward from the value of pressure at point P$_{3}$ to check the accuracy in reproducing the experimentally obtained pressure at point P$_{2}$. The final profile is calculated by averaging the forwardly and backwardly iterated pressures at each available centreline position along the Venturi meter. Images were recorded with a high-speed Phantom Speedsense 9040 camera placed in front of the Venturi tube with an image resolution of $1260\times 1024$ pixels and working at 1000 frames per second. The velocity profiles were obtained by seeding the water with polyamide spherical tracer particles of 50 nm diameter each. The Dantec Dynamic Studio software was employed to process the acquired images.

The experiments were performed at an ambient temperature of $30\,^{\circ }$C for volumetric flow rates between 10 and 25 lpm, corresponding to bulk velocities at the inlet of the Venturi meter $v_{\text{b}}=1.05$ and 2.625 m s$^{-1}$, respectively. At such flow rates, the flow through the Venturi meter corresponds to Reynolds numbers, Re$=\rho v_{\text{b}}D_{\text{H}}/\mu$, between 13272 (for $Q=10$ lpm) and 33180 (for $Q=25$ lpm), where $\mu =0.000798$ kg m$^{-1}$ s$^{-1}$ is the dynamic viscosity of water at $30\,^{\circ }$C.

### Governing equations

The differential equations describing the flow of water through the Venturi meter are given by the Navier-Stokes equations

1 $$\begin{aligned} \frac{d\rho }{dt}=  {} -\rho \nabla \cdot \textbf{v}, \end{aligned}$$

2 $$\begin{aligned} \frac{d\textbf{v}}{dt}=  {} -\frac{1}{\rho }\nabla p-\nu \nabla ^{2}{} \textbf{v}+ \textbf{g}, \end{aligned}$$

where $\rho$ is the mass density, $\textbf{v}$ is the velocity vector, *p* is the pressure, $\nu$ is the coefficient of kinematic viscosity, $\textbf{g}$ is the gravitational acceleration and $d/dt=\partial /\partial t+\textbf{v}\cdot \nabla$ is the material time derivative. The pressure is defined in terms of the density using the Murnaghan-Tait equation of state^34^

3 $$\begin{aligned} p=p_{0}\left[ \left( \frac{\rho }{\rho _{0}}\right) ^{\gamma }-1\right] , \end{aligned}$$

where $\gamma =7$ is appropriate for water, $p_{0}=c_{0}^{2}/\gamma$, $\rho _{0}=995.71$ kg m$^{-3}$ is the water density at $30\,^{\circ }$C and $c_{0}$ is the sound speed at the reference density $\rho _{0}$. The kinematic viscosity of water at $30\,^{\circ }$C is $\nu =8.01\times 10^{-7}$ m$^{2}$ s$^{-1}$. Here we adopt a weakly compressible approach and the term $p_{0}$ governs the relative density fluctuations $|\rho -\rho _{0}|/\rho _{0}\sim M^{2}$, where the Mach number *M* is typically set to 0.01 in order to enforce density fluctuations less than about 1%. This is accomplished by defining the reference sound speed $c_{0}$ to be at least ten times higher than the maximum fluid velocity across the flow meter.

### Numerical methods

In the SPH framework a LES filtering of Eqs. (1) and (2) coupled with a sub-particle scaling technique is implemented to model coherent turbulent structures in the fluid^39^. In this approach, the velocity field is separated into its mean component, $\tilde{\textbf{v}}$, and its fluctuating part, $\textbf{v}^{\prime }$, such that $\textbf{v}=\tilde{\textbf{v}}+\textbf{v}^{\prime }$, where the mean velocity is obtained by means of the density-weighted Favre-filtering

4 $$\begin{aligned} \tilde{\textbf{v}}=\frac{1}{{\bar{\rho }}}\frac{1}{T}\int _{t}^{t+T}\rho (\textbf{x},t) \textbf{v}(\textbf{x},t)dt, \end{aligned}$$

where *T* is a sufficiently large time interval and ${{\bar{\rho }}}$ is the Reynolds-averaged density. After application of the Favre-filtering, Eqs. (1) and (2) become

5 $$\begin{aligned} \frac{d{{\bar{\rho }}}}{dt}=  {} -{{\bar{\rho }}}\nabla \cdot \tilde{\textbf{v}}, \end{aligned}$$

6 $$\begin{aligned} \frac{d\tilde{\textbf{v}}}{dt}=  {} -\frac{1}{{\bar{\rho }}}\nabla {\tilde{p}}+ \frac{\nu }{{\bar{\rho }}}\left[ \nabla \cdot \left( {{\bar{\rho }}}\nabla \right) \right] {\tilde{\textbf{v}}} +\frac{1}{{\bar{\rho }}}\nabla \cdot {\mathbb {T}}+\textbf{g}. \end{aligned}$$

Here, $\mathbb {T}$ is the sub-particle stress tensor

7 $$\begin{aligned} T_{ij}={{\bar{\rho }}}\nu _{t}\left( 2S_{ij}-\frac{2}{3}S_{kk}\delta _{ij}\right) - \frac{2}{3}{{\bar{\rho }}}C_{I}\Delta ^{2}\delta _{ij}|S|^{2}, \end{aligned}$$

where

8 $$\begin{aligned} S_{ij}=\frac{1}{2}\left( \frac{\partial {\tilde{v}}_{i}}{\partial x_{j}}+ \frac{\partial {\tilde{v}}_{j}}{\partial x_{i}}\right) , \end{aligned}$$

is the Favre-filtered strain rate tensor, $C_{I}=0.00066$, $\nu _{t}=(0.12\nabla )^{2}|S|$ is the Smagorinsky eddy viscosity, $|S|=(2S_{ij}S_{ij})^{1/2}$ is the local strain rate, $\delta _{ij}$ is the Kronecker delta and $\Delta$ is a measure of the finite particle size.

Equations (5) and (6) are solved numerically using a modified version of DualSPHysics^33^, which is based on SPH methods^40,41^. SPH is a fully Lagrangian, mesh-free scheme for the simulation of complex fluid-flows, where the fluid is represented by a discrete number of moving particles that carry all field information of the system as, for example, the density and velocity. In DualSPHysics the density of particle *a* is calculated using Eq. (5), which in discrete form is written as

9 $$\begin{aligned} \frac{d\rho _{a}}{dt}=\sum _{b=1}^{n}m_{b}\left( \textbf{v}_{a}-\textbf{v}_{b} \right) \cdot \nabla _{a}W_{ab}, \end{aligned}$$

where $\rho _{a}$ is the particle-scale density associated to particle *a*, $m_{b}$ is the mass of neighboring particle *b*, $W_{ab}=W(|\textbf{x}_{a}-\textbf{x}_{b}|,h)$ is the kernel function, *h* is the smoothing length and *n* is the number of neighbors of particle *a* within the kernel support, i.e., within distances $|\textbf{x}_{a}-\textbf{x}_{b}|\le h$ from particle *a*. The value of *n* is chosen to be $n=0.12\sqrt{N}$, where *N* is the total number of SPH particles filling the computational domain. This prescription complies with the joint limit $N\rightarrow \infty$, $n\rightarrow \infty$ and $h\rightarrow 0$ for complete convergence to the continuum^37,38^. The SPH form of Eq. (6) is given by

10 $$\begin{aligned} \frac{d\textbf{v}_{a}}{dt} &= {} -\frac{1}{\rho _{a}}\sum _{b=1}^{n} \frac{m_{b}}{\rho _{b}}\left( p_{a}+p_{b}\right) \nabla _{a}W_{ab} +4\nu \sum _{b=1}^{n}m_{b}\frac{\textbf{v}_{a}-\textbf{v}_{b}}{\rho _{a}+ \rho _{b}}\frac{\textbf{x}_{ab}\cdot \nabla _{a}W_{ab}}{|\textbf{x}_{ab}|^{2}+\varepsilon ^{2}}\nonumber \\{} &\quad {} +\sum _{b=1}^{n}m_{b}\left( \frac{\mathbb {T}_{a}}{\rho _{a}^{2}}+ \frac{\mathbb {T}_{b}}{\rho _{b}^{2}}\right) \cdot \nabla _{a}W_{ab}+\textbf{g}, \end{aligned}$$

where $\textbf{x}_{ab}=\textbf{x}_{a}-\textbf{x}_{b}$ and $\varepsilon ^{2}=0.01h^{2}$. In the above SPH representations the bar and tilde operators over the mean density and ensemble average velocity vector have been dropped for simplicity.

The particles are moved along the Venturi meter by integrating the equation

11 $$\begin{aligned} \frac{d\textbf{x}_{a}}{dt}=\textbf{v}_{a}+\frac{\beta x_{0}v_{\text{max}}}{M} \sum _{b=1}^{N}m_{b} \frac{\textbf{x}_{ab}}{\left( \textbf{x}_{ab}\cdot \textbf{x}_{ab}\right) ^{3/2}}, \end{aligned}$$

where the second term on the right-hand side prevents the growth of errors due to anisotropies in the distribution of particle positions^42^. Here $\beta =0.04$, $v_{\max }$ is the maximum velocity, *M* is the total fluid mass within the computational domain and $x_{0}$, given by

12 $$\begin{aligned} x_{0}=\frac{1}{N}\sum _{b=1}^{N}\left( \textbf{x}_{ab} \cdot \textbf{x}_{ab}\right) ^{1/2}, \end{aligned}$$

is the mean distance between particle *a* and all other particles, where the summation is taken over all *N* particles filling the computational domain. The convergence properties of SPH are improved by adopting a C$^{2}$ Wendland function as the interpolation kernel^35,36^

13 $$\begin{aligned} W(q,h)=\frac{21}{2\pi h^{3}}\left( 1-q\right) ^{4}\left( 1+4q\right) , \end{aligned}$$

for $q\le 1$ and zero otherwise, where $q=|\textbf{x}-\textbf{x}^{\prime }|/h$. The time integration of Eqs. (9)–(11) is performed using the Verlet algorithm provided by DualSPHysics, which is second-order accurate and maintains adequate numerical coupling among Eqs. (9)–(11) during the evolution. The use of Eq. (3) enforces a weakly compressible SPH scheme. At small scales most fluids are incompressible and an alternative SPH approach that satisfies this condition is the so-called incompressible SPH^43^. This approach has been successfully applied in the simulation of free-surface flows, including Newtonian and non-Newtonian flows, dam break flows and wave overtopping on different coastal structures^44–47^, among many other applications.

The numerical integration of Eqs. (9)–(11) is performed by applying no-slip boundary conditions (i.e., $\textbf{v}=\textbf{0}$) at the solid walls of the Venturi tube using the method of dynamic boundary particles proposed by Crespo et al.^48,49^. In these simulations, a layer of particles at rest defines the walls of the Venturi and two layers of ghost particles are placed outside the computational domain around the volume occupied by the Venturi meter. Although these particles are updated using Eq. (10), they are not allowed to move so they preserve their initial positions. The density of the outer ghost particles is determined from the neighboring fluid particles lying within their support domains. Particle penetration across the solid walls is avoided by allowing the wall particles to exert repulsive forces on nearby fluid particles, which are derived from the source of Eq. (10).

**Figure 3:**
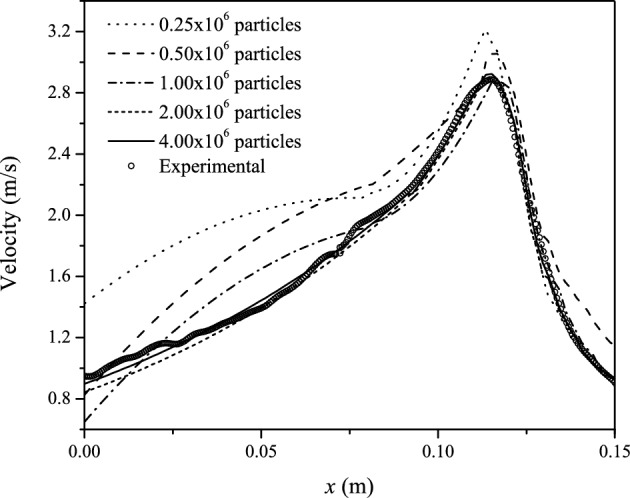
Centerline velocity profiles along the Venturi meter for $Q=10$ lpm at different spatial resolutions as compared to the experimental data. Asymptotic convergence to the experimental data is already observed for $N\ge 2015768$ SPH particles. For reference $x=0.15$ m and $x=0$ m mark the positions of the entrance (L$_{1}$) and exit (L$_{5}$) planes of the Venturi meter, respectively.

Open boundary conditions are applied at the inlet, L$_{1}$, and outlet, L$_{5}$, planes (see Fig. 2). An inflow zone is placed in front of the inlet plane, where inflow particles are arranged regularly and allowed to flow in as needed with prescribed density and velocity values^50^. Non-reflecting outflow boundary conditions are implemented by defining an outflow zone in front of the outlet (L$_{5}$) plane such that particles there are moved using an outgoing wave equation of the form

14 $$\begin{aligned} \frac{\partial \textbf{v}}{\partial t}+ v_{x}\frac{\partial \textbf{v}}{\partial x} -\nu \left( \frac{\partial ^{2}{} \textbf{v}}{\partial y^{2}}+ \frac{\partial ^{2}{} \textbf{v}}{\partial z^{2}}\right) =\textbf{0}, \end{aligned}$$

where $\textbf{v}=(v_{x},v_{y},v_{z})$ and the mainstream velocity across the L$_{5}$-plane is assumed to be along the *x*-axis. A numerically stable SPH representation of this equation can be written as

15 $$\begin{aligned} \frac{\partial \textbf{v}_{o}}{\partial t}=-v_{x,o}\sum _{b=1}^{n}\frac{m_{b}}{{{\bar{\rho }}}_{ob}}\left( \textbf{v}_{b}-\textbf{v}_{o}\right) \frac{\partial W_{ob}}{\partial x_{o}} +2\nu \sum _{b=1}^{n}\frac{m_{b}}{\rho _{b}} \frac{\left( \textbf{v}_{b}-\textbf{v}_{o}\right) }{|\textbf{x}_{ob}|^{2}+\varepsilon ^{2}} \left( y_{ob}\frac{\partial W_{ob}}{\partial y_{o}}+z_{ob} \frac{\partial W_{ob}}{\partial z_{o}}\right) , \end{aligned}$$

where the subscript *o* is used to denote particles in the outflow zone. Here $\textbf{x}_{ob}=\textbf{x}_{o}-\textbf{x}_{b}$, $y_{ob}=y_{o}-y_{b}$, $z_{ob}=z_{o}-z_{b}$ and ${{\bar{\rho }}}_{ob}=(\rho _{o}+\rho _{b})/2$. Since the inlet and outlet mass rates may differ from each other, a particle leaving the outflow zone is temporarily stored in a reservoir buffer. As an inflow particle crosses the L$_{1}$-plane, a particle is removed from the buffer and placed in the upstream side of the inflow zone with a prescribed density and velocity. A more stable scheme with the use of Eq. (15) can be obtained by smoothing the mainstream velocity component, $v_{x,o}$, according to the prescription

16 $$\begin{aligned} v_{x,o}=\sum _{b=1}^{n}\frac{m_{b}}{\rho _{b}}v_{x,b}W_{ob}, \end{aligned}$$

where a particle *o* can have neighbors pertaining to the fluid and outflow zones depending on how close it is from the outlet plane. Finally, the position and velocity of outflow particles is obtained by integrating the equation

17 $$\begin{aligned} \frac{d\textbf{x}_{o}}{dt}=\textbf{v}_{o}, \end{aligned}$$

simultaneously with Eq. (15) using the same Verlet integrator employed to evolve the hydrodynamics.

## Results

### Convergence testing and numerical validation

A resolution independence test was performed for the SPH simulations to determine the quality of the numerical solutions and convergence to the experimental data. The experimentally obtained mainstream velocity variation along the Venturi meter for a volumetric flow rate of 10 lpm was chosen as a convergence test for numerical validation purposes. The SPH particles are initially at rest and uniformly distributed in all three coordinate directions so that $\Delta =\Delta x=\Delta y=\Delta z$ and the smoothing length is set equal to $C\sqrt{3}\Delta$, with $C=1.15$, for all runs. Figure 3 shows the SPH results as the number of particles is increased from $N=254288$ to 4054963 particles. The numerical profiles show a tendency to globally converge to the experimental data (open circles) as the spatial resolution is improved. The last two columns of Table 1 list the maximum velocity at the exit of the throat section ($x=0.115$ m) and the root-mean-square errors (RMSEs) between the numerical and experimental profiles at different resolutions, respectively. In terms of this metric, the error between the SPH and the experimental data decays from $\approx 0.41$ m s$^{-1}$ for $N=254288$ particles (with more significant deviations in the Venturi diffuser) to $\approx 0.04$ m s$^{-1}$ for $N=4054963$ particles (see Table 1). At the highest resolution the numerical profile fits the experimental one with only an error of $\approx 4.3$%. The position and magnitude of the experimental peak velocity ($\approx 2.887$ m s$^{-1}$) at $x=0.115$ m is very well reproduced for $N\ge 2015768$ particles, with values of $\approx 2.89$ m s$^{-1}$ (for $N=2015768$) and $\approx 2.92$ m s$^{-1}$ (for $N=4054963$). The higher dispersion of the SPH velocity profiles along the diffuser ($x<0.075$ cm) for $\le 1$ million particles occurs because near the pipe walls the SPH velocity values are larger than the real ones, resulting in more pronounced parabolic profiles than it should be. As the number of particles is increased, the flow velocity near the walls becomes closer to the experimental measurements and the dispersion is drastically reduced.

**Table 1:** Spatial resolution parameters, maximum velocity and RMSEs for the convergence test.

Number of SPH particles *N*	Number of neighbors *n*	Smoothing length *h* (mm)	Maximum velocity (m s^−1^)	RMSE (m s^−1^)
531404	88	1.295	3.06	0.278
1056253	123	0.996	2.88	0.141
2015768	171	0.797	2.89	0.068
4054963	242	0.637	2.92	0.043

**Figure 4:**
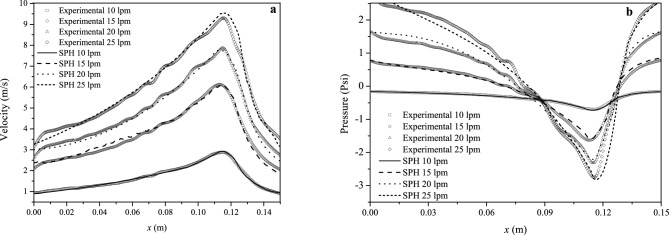
(**a**) Centerline mainstream velocity and (**b**) pressure profiles along the Venturi meter for varying volumetric flow rates between $Q=10$ and 25 lpm as compared with the experimental data.

### Comparison with the experimental data

Figure 4a,b show the centerline velocity and pressure profiles along the Venturi meter for $Q=10$, 15, 20 and 25 lpm. In each figure the position $x=0.25$ m marks the entrance plane (L$_{1}$), while $x=0$ m marks the exit plane (L$_{5}$) of the Venturi tube. The last two columns of Table 2 list the RMSEs between the experimental and SPH velocity and pressure profiles, respectively. These errors increase with increasing volumetric flow rate. For the velocity, the SPH simulations reproduce the experimental data with $\approx 4.3$% for $Q=10$ lpm and $\approx 7.1$% for $Q=25$ lpm. When measured in units of psi, the pressure profiles are reproduced with RMSEs that vary between $\approx 1.4$% for $Q=10$ lpm to about 6.8% for $Q=25$ lpm. The RMSE between the experimentally measured and the numerically obtained differential pressure also increases with the volumetric flow rate.

**Table 2:** Flow characteristics and errors of centerline mainstream velocity and pressure profiles along the Venturi meter.

Volumetric flow rate *Q* (lpm)	Bulk velocity $v_{\text{b}}$ (m s^−1^)	Mass flow $\dot{m}$ (kg s^−1^)	RMSE (*v*) (m s^−1^)	RMSE (*p*) (Psi)
10	1.050	0.17	0.043	0.014
15	1.575	0.25	0.054	0.034
20	2.100	0.33	0.063	0.056
25	2.625	0.42	0.071	0.068

**Figure 5:**
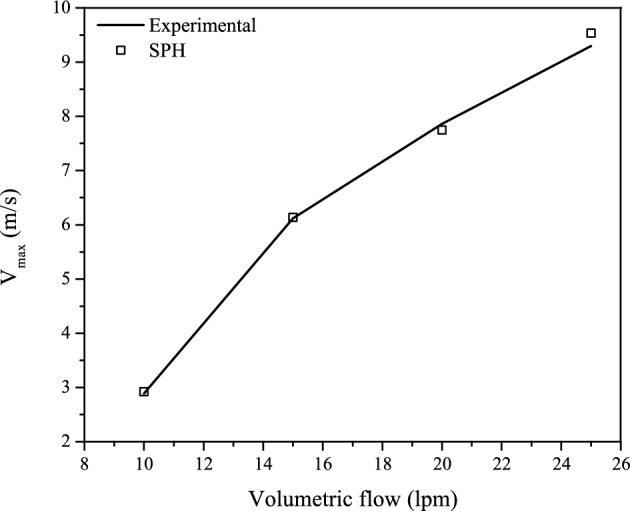
Experimentally measured maximum flow velocities at the exit of the throat section (solid line) as compared with the numerically obtained values (blank squares).

**Figure 6:**
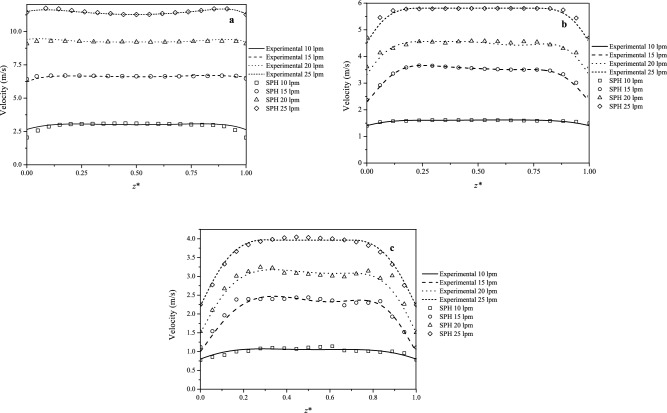
Numerical horizontal mainstream velocity profiles for varying volumetric flow rates between $Q=10$ and 25 lpm as compared with the experimental data at pipe stations: (**a**) L$_{3}$ within the throat section, (**b**) L$_{4}$ in the middle of the divergent section and (**c**) L$_{5}$ at the exit plane of the divergent section.

**Figure 7:**
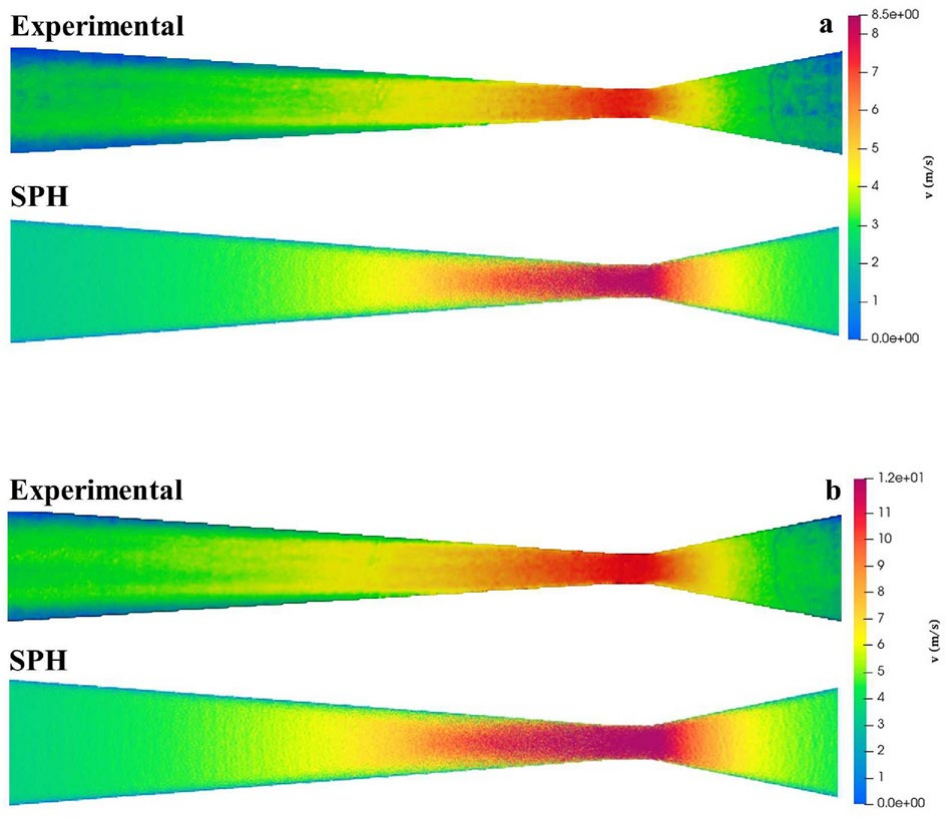
Comparison between the experimental and SPH mainstream velocity maps along the Venturi meter for (**a**) 15 lpm and (**b**) 25 lpm.

The experimentally observed position of the maximum velocity and pressure at the exit of the throat section ($x\approx 0.115$ m) is very well reproduced by the SPH simulations. The experimental and SPH maximum velocities in units of m s$^{-1}$ are ($v_{\text{max,exp}}$,$v_{\text{max,SPH}}$)=(2.89,2.92) for $Q=10$ lpm, (6.12,6.14) for $Q=15$ lpm, (7.86,7.75) for $Q=20$ lpm, and (9.30,9.53) for $Q=25$ lpm. These figures show that the strength of the peak velocities is fairly well reproduced by the SPH simulations. The largest differences are observed for $Q=20$ and 25 lpm. The numerically obtained maximum velocities as compared to the experimental data are depicted in Fig. 5 for all volumetric flow rates considered. In units of psi, the experimental and SPH minimum pressure values at the exit of the throat section are: ($p_{\text{min,exp}}$,$p_{\text{min,SPH}}$)=($-0.70$,$-0.72$) for $Q=10$ lpm, ($-1.63$,$-1.57$) for $Q=15$ lpm, ($-2.32$,$-2.16$) for $Q=20$ lpm, and ($-2.75$,$-2.88$) for $Q=25$ lpm.

The mainstream velocity profiles in the horizontal (*z*) plane (see Fig. 1), with $z^{\star }=z/(6.35$ mm), as compared with the experimental measurements are shown in Fig. 6 at pipe stations (a) L$_{3}$, (b) L$_{4}$ and (c) L$_{5}$ (see Fig. 2), respectively. Each figure depicts the profiles for $Q=10$, 15, 20 and 25 lpm. The experimental measurements were repeated several times for reproducibility and so they are considered to be accurate enough to provide a useful benchmark test case for checking the accuracy of the SPH flow prediction method. In all cases the SPH profiles closely match the experimental data with RMSE deviations ranging from $\approx 2.1$% at L$_{3}$ for $Q=10$ lpm to about 5.5% at L$_{4}$ for $Q=20$ lpm (see Table 3). As the core of the vena contracta expands through the diffuser, the velocity towards the pipe walls decays less steeply consistently with the flow there approaching a parabolic shape.

**Table 3:** RMSE deviations between the experimental and SPH cross-sectional mainstream velocity profiles.

Pipe station	$Q=10$ lpm	$Q=15$ lpm RMSE(*v*) (m s$^{-1}$)	$Q=20$ lpm	$Q=25$ lpm
L$_{3}$	0.021	0.029	0.036	0.051
L$_{4}$	0.027	0.034	0.041	0.055
L$_{5}$	0.035	0.038	0.047	0.053

Table 3 lists the RMSE deviations between the experimental and SPH cross-sectional mainstream velocity profiles for all volumetric flow rates considered. The errors increase with increasing values of *Q*. At pipe station L$_{3}$, within the throat section, the profiles are almost flat with very sharp decays to zero close to the pipe walls. As *Q* is increased the extension of the vena contracta also increases and for $Q=25$ lpm it becomes as long as the divergent section. This explains the almost flat profile in the central core with less steep decays to zero towards the pipe walls for $Q\ge 15$ lpm. In particular, for $Q=10$ lpm the vena contracta downstream of the throat exit shortens and the flow becomes almost flat, though at a lower velocity compared to station L$_{3}$. Close to the exit of the diffuser (station L$_{5}$), the vena contracta weakens and the flow try to revert to laminar. However, a smaller fraction of the central core still remains approximately flat. Therefore, as the vena contracta completely diffuses outside the Venturi tube the profile gradually approaches a parabolic shape. Figure 7 shows a direct comparison between the experimental and the SPH calculated mainstream velocity field along the Venturi meter for (a) 15 lpm and (b) 25 lpm. The velocity fields are qualitatively similar with small differences close to the inlet and outlet of the Venturi tube.

## Conclusions

In this study a modified version of the open-source code DualSPHysics, which relies on the method of Smoothed Particle Hydrodynamics (SPH), has been used to simulate the flow of water at moderately high Reynolds numbers through a horizontal, small-scale Venturi meter of rectangular cross-sectional shape. The geometry and dimensions of the Venturi tube as well as the initial flow parameters used for the numerical simulations correspond to the experimentally defined values.

As the flow rate is increased within the allowed experimental range, the SPH simulations predict mainstream velocity profiles and differential pressures along the Venturi tube that are in good qualitative agreement with the experimental data. The deviations between the SPH and the experimental mainstream velocity profiles vary from 4.3% for a volumetric flow rate (Q) of 10 liters per minute (lpm) to 7.1% for $Q=25$ lpm, while the SPH differential pressures differ from the experimental data by about 1.4% (for $Q=10$ lpm) and 6.8% (for $Q=25$ lpm). The cross-sectional velocity profiles within the throat and divergent sections are always less than 5.5%. In general, the largest deviations from the experimental data occur for $Q=25$ lpm. At higher flow rates, additional experiments have shown that cavitating flows may form within the throat section due to much stronger pressure losses. As a further step towards improving the quality of the SPH simulations for calibrating laboratory-scale and industrial-size flow metres, the results from this study will be extended to simulate higher flow rates and include thermal effects through the use of an internal energy equation at much higher spatial resolutions to allow for direct numerical simulations. The present results have applications in deep water culture (DWC) systems^51^. Section varying devices with square or rectangular cross-section are common implementations in controlled irrigation systems within the schemes of precision farming as in open-loop techniques and DWC, due to the square geometry of flooded receptacles for plants. As a low-impact tool, the Venturi meter studied here can be added to the output of the pumping system to inject air into the pond which can aid in keeping high dissolved oxygen levels.

## Data Availability

Data will be made available from the corresponding author upon request.
